# Women’s experiences and perceptions of postpartum care in Saudi Arabia: a qualitative study

**DOI:** 10.1186/s12884-026-08919-3

**Published:** 2026-03-09

**Authors:** Fatimah Almalki, Noura Alomair

**Affiliations:** 1 Security Forces Hospitals Program, General Directorate of Medical Services, Ministry of Interior, Riyadh, Saudi Arabia; 2 Community Health Sciences Department, College of Applied Medical Sciences, King Saud University, Riyadh, Kingdom of Saudi Arabia

**Keywords:** Postpartum care, Qualitative research, Saudi Arabia, Women’s experiences, WHO guidelines, Pender’s Health Promotion Model

## Abstract

**Background:**

Postpartum care is essential for maternal well-being, yet limited qualitative work in Saudi Arabia has explored how women make sense of early postnatal experiences. This study explored postpartum women’s experiences of birth and hospital care and how perceived emotional, informational, and cultural support shaped recovery and health-promoting behaviours.

**Methods:**

A qualitative study guided by Reflexive Thematic Analysis using semi-structured interviews was conducted at King Khaled University Hospital in Riyadh. Data were collected between January and February 2025. Purposive sampling was used to recruit 20 postpartum women who were medically stable, had recently given birth (vaginal or caesarean), had different levels of parity and were able to participate in an interview.

**Results:**

Four interconnected themes described women’s experiences: physical recovery and birth experiences; informational and educational support; emotional and social support; and systemic and cultural influences. Women identified gaps in several WHO-recommended elements, including consistency of pain management, breastfeeding counselling, and discharge preparation. Limited informational support reduced perceived control and hindered engagement in health-promoting behaviours, reflecting key constructs of Pender’s model. Emotional well-being was shaped by the quality of staff communication, perceived respect, and the availability of family support, which influenced confidence and adjustment.

**Conclusion:**

This study highlights that, although clinical care is generally competent, gaps in emotional support, informational continuity, nursery availability, and discharge preparedness continue to affect women’s confidence, recovery, and perceived safety. Strengthening postpartum services in line with WHO postnatal care standards through flexible, woman-centred, and culturally responsive approaches together with enhanced behavioural support may improve maternal adaptation and overall well-being. Future multi-site research is needed to guide postpartum care policies and service models more broadly in Saudi Arabia.

## Background

 Postpartum or postnatal care refers to the comprehensive medical, emotional, and educational support provided to mothers and newborns after childbirth [1]. Although this period involves major physiological and psychological adjustments, such as breastfeeding initiation, hormonal changes, and heightened vulnerability to complications [2], women’s emotional transitions, expectations equally define the postpartum phase, and the quality of support they receive. The World Health Organisation (WHO) recommends at least three postnatal visits within the first six weeks [1]; however, global adherence remains inconsistent due to differences in health-system readiness, sociocultural beliefs, family support structures, and maternal awareness of postpartum needs [3]. Globally, up to one in five women experience postpartum mental health concerns, which often remain undetected in settings without systematic follow-up or routine psychosocial screening [4–6]. These global disparities reflect challenges similar to those reported in several Middle Eastern contexts, including Saudi Arabia, where access, continuity, and psychosocial aspects of postpartum care remain variably addressed.

Health systems worldwide have increasingly recognised that postpartum care must extend beyond clinical monitoring to include holistic, woman-centred support. This shift reflects a broader understanding that childbirth and the postpartum period are deeply personal experiences shaped by cultural norms, social expectations, marital and familial dynamics, and interactions with healthcare providers [1, 2]. Women’s postpartum experiences involve not only physical recovery but also evolving identities, emotional vulnerability, empowerment, confidence-building, and the need for clear, compassionate, and culturally respectful information. In this study, “experience” refers to how women interpret and emotionally navigate postpartum care, while “perception” reflects how they evaluate, make meaning of, and form expectations about the care they receive. Subjective experiences are increasingly recognised as essential indicators of quality, influencing maternal satisfaction, care engagement, breastfeeding continuation, and mental well-being [7]. 

Across countries, the organisation and delivery of postpartum services vary significantly. High-income settings such as the United Kingdom, Sweden, and Australia have integrated postpartum care pathways that include breastfeeding counselling, mental health screening, home visits, and continuity-of-care models approaches shown to improve maternal outcomes and reduce preventable complications [1, 8]. Conversely, low- and middle-income countries commonly face workforce shortages, limited postnatal follow-up, and reliance on informal or traditional care practices. In many regions, mothers encounter gaps in emotional support, inconsistent breastfeeding counselling, and limited access to psychosocial services, which can hinder recovery and delay help-seeking [9]. Understanding these variations is essential for contextualising postpartum experiences in Saudi Arabia, where service availability, cultural norms, and expectations of support differ from global patterns.

In Saudi Arabia, maternal healthcare is delivered through both governmental and private sectors. Public hospitals and primary care centres provide free maternity services in line with national guidelines aligned with international standards, while private facilities complement these services. Initiatives such as the Mother-and-Baby-Friendly Childbirth Initiative aim to promote evidence-based and respectful maternity care, enhance mother–infant bonding, and strengthen postpartum support [10, 11]. Despite these advancements, existing research in Saudi Arabia remains predominantly quantitative, focusing on clinical outcomes, service utilisation, or specific medical indicators. Qualitative research that centres mothers’ voices, lived experiences, cultural expectations, and emotional needs remains limited. Evidence from recent regional studies suggests that women’s perspectives are still underrepresented in evaluations of postpartum care, highlighting a crucial gap in understanding the relational and contextual factors that shape their experiences [12].

This study was informed by two complementary frameworks relevant to postpartum care. The World Health Organization (WHO) recommendations on maternal and newborn care for a positive postnatal experience (2022), hereafter referred to as the WHO postnatal care (PNC) guidelines, outline expected standards of physical, informational, and psychosocial support during the postpartum period [1], while Pender’s Health Promotion Model (HPM) emphasises behavioural, interpersonal, and situational factors influencing engagement in health-promoting behaviours [13]. Guided by these frameworks, the study aimed to explore women’s emotional, informational, social, and clinical experiences of postpartum care, with particular attention to communication, education, and the cultural and social factors shaping postpartum recovery.

## Methods

### Study design

This study adopted a reflexive qualitative design underpinned by an interpretivist paradigm, which assumes that reality is socially constructed and that meanings are shaped through individuals’ experiences, contexts, and interactions [14]. Reflexive Thematic Analysis, as described by Braun and Clarke, was used to explore how postpartum women make sense of their care experiences [15]. Semi-structured interviews enabled in-depth exploration of women’s subjective interpretations while ensuring comprehensive coverage of core postpartum care topics.

### Study setting

The study was conducted at King Khalid University Hospital (KKUH), a tertiary teaching hospital affiliated with King Saud University Medical City in Riyadh, Saudi Arabia. KKUH was selected because it serves a diverse population and provides comprehensive maternity services, making it an appropriate setting for examining varied postpartum experiences. Data collection occurred between January 15 and February 16, 2025, within the postnatal units.

### Sampling and recruitment strategy

A purposive sampling strategy was employed to recruit postpartum women who had given birth either vaginally or via caesarean section within the preceding 16–96 h, were medically stable, able to participate, and had provided written informed consent. Women were excluded if they experienced severe medical complications that hindered participation or if they declined or withdrew consent.

Participants were initially identified with assistance from nursing staff, who generated a list of eligible mothers. The first author (FA) approached mothers in person, explained the study aims and procedures, and invited them to participate. Variation in age, nationality, parity, and mode of delivery was sought to ensure diversity in postpartum perspectives. There was no prior relationship between the researcher and participants before recruitment; no participants withdrew from the study. A total of 20 women completed interviews, providing adequate information for in-depth thematic analysis.

Recruitment continued until data saturation was reached, defined as the point at which no new codes or concepts emerged during analysis. Sample adequacy was further supported by sufficient information power, given the specificity of the sample, the focused study aim, and the richness of the interview data [16].

### Interview procedures

Semi-structured interviews were conducted in the postnatal care units of KKUH, either in private or shared rooms, depending on room allocation. Sixteen interviews were conducted in private rooms. Four interviews took place in shared rooms due to unit occupancy; in these cases, privacy was maintained by fully drawing the curtains around the participant’s bed, positioning the interviewer in proximity, and conducting the conversation in a low, discreet voice to minimise the risk of being overheard.

Eleven participants had a companion present (e.g., a mother, sister, husband, or domestic helper), a common practice in Saudi postnatal settings. Companions were informed about the interview and were asked not to participate in or influence the conversation. All participants provided verbal and written consent to proceed despite the presence of a companion. No companion intervened or contributed to the interview, and the interviewer monitored the dynamics closely to ensure that participants’ responses reflected their own experiences and perspectives.

The interviews explored a range of topics, including birth experiences, maternal adjustment, informational needs, inpatient care, discharge preparation, emotional well-being, cultural influences, and suggestions for service improvement. The interview guide was pilot-tested with four postpartum mothers, and revisions included clarifying culturally sensitive questions, improving topic sequencing to ensure smoother flow, and strengthening probes to address emotional, informational, and support needs. All interviews were conducted by the first author (FA), a female Health Education and Promotion specialist with formal training in qualitative interviewing. Interviews lasted 30–45 min. No repeat interviews were conducted.

### Data collection and management

With informed consent, all interviews were audio recorded and supplemented by field notes capturing nonverbal cues and contextual observations. A brief socio-demographic questionnaire collected age, nationality, education, employment, parity, and mode of delivery.

Nineteen interviews were conducted in Arabic and one in English. All audio files were transcribed verbatim. All Arabic transcripts were translated into English by the first author and subsequently reviewed by a bilingual co-author to ensure accuracy, cultural equivalence, and preservation of meaning across languages. Transcripts were not returned to participants, consistent with reflexive thematic analysis, which views meaning-making as interpretive rather than a process of participant verification. All data were anonymised and stored on password-protected, access-restricted devices in accordance with institutional ethics protocols.

### Data analysis

Data were analysed using Reflexive Thematic Analysis (RTA) following Braun and Clarke’s six phases [15, 17]. Transcripts were read repeatedly, and initial codes were generated inductively. Coding and theme development were iterative and reflexive, with analytic decisions documented throughout. Initial codes were progressively grouped into subcategories and refined into final themes, forming the study’s analytical coding structure. ATLAS.ti 23 software supported the organisation, management, and retrieval of codes and analytic memos.

Two researchers (FA and NA) independently coded a subset of transcripts. Differences in interpretation were treated as opportunities for deeper reflection, discussed collaboratively, and resolved through discussions, consistent with RTA principles [18].

**Fig. 1:**
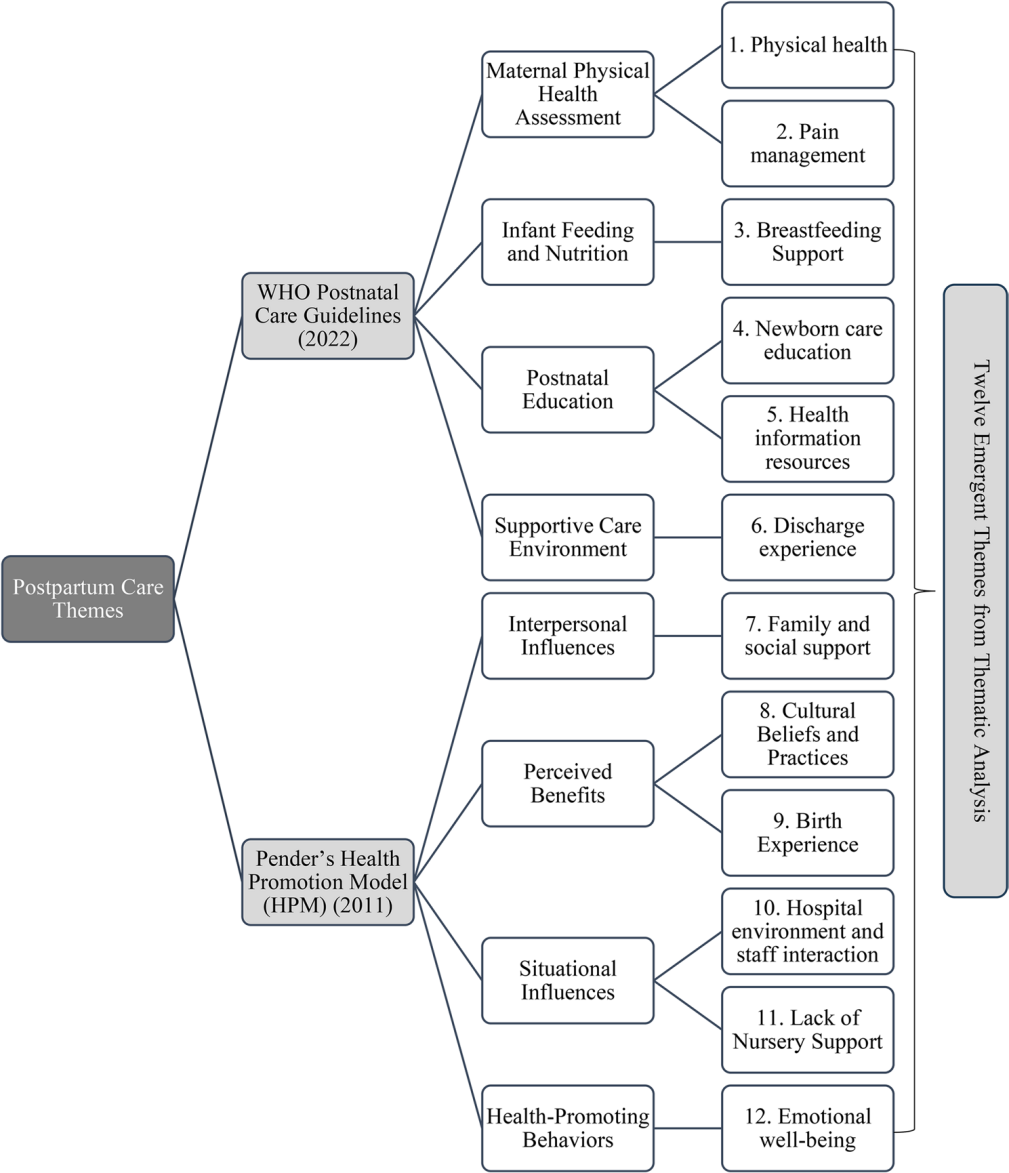
Mapping of postpartum care themes to WHO Postnatal Care Guidelines [1] and Pender’s Health Promotion Model [13].

Coding was primarily inductive, with initial themes generated from participants’ narratives. The WHO PNC guidelines and HPM were subsequently applied as interpretive lenses during later stages of analysis to contextualise and deepen understanding of the findings. These frameworks were not used to generate codes; rather, they informed analytic interpretation by clarifying the relational patterns between themes within established postpartum care domains and behavioural theory. The WHO PNC guidelines [1] specifically informed interpretations related to breastfeeding support, physical recovery, newborn care education, discharge preparation, and access to reliable health information. In parallel, HPM [13] guided the analysis of interpersonal influences, perceived benefits, situational barriers, and behavioural responses shaping women’s postpartum adaptation. To visually demonstrate how emergent themes aligned with these frameworks, a conceptual map was developed (Fig. 1), illustrating the correspondence between participants’ narratives and key domains of the WHO PNC guidelines and HPM.

A reflexive stance was maintained throughout the analytic process, acknowledging how the researchers’ professional backgrounds in women’s health and public health could shape interpretation. Reflexive journaling and regular debriefing discussions were used to enhance transparency, minimise bias, and strengthen the credibility of the findings. Participant checking was not conducted, in line with the reflexive thematic analysis approach, which conceptualises meaning as co-constructed through researcher interpretation rather than participant validation.

### Trustworthiness

Several strategies were employed to enhance the trustworthiness of the study. Credibility was supported by close, iterative engagement with the data, reflexive coding, and collaborative discussions between authors throughout the analytic process. Confirmability was enhanced through reflexive journaling and maintenance of an audit trail documenting key analytic decisions. Dependability was addressed through detailed documentation of data collection and analysis procedures, ensuring methodological transparency and consistency. Transferability was strengthened by providing rich contextual descriptions of the study setting, participants, and cultural context, enabling readers to assess the applicability of the findings to similar contexts.

### Ethical considerations

Ethical approval was obtained from the Institutional Review Board at KKUH (E-24-9376) and written official approval from the Nursing Research Unit at KKUH. All participants received written and verbal information about the study and signed informed consent forms. Ethical principles of autonomy, beneficence, and confidentiality were strictly maintained throughout the study. The reporting of this study adhered to the COREQ (Consolidated Criteria for Reporting Qualitative Research) guidelines to ensure transparency and completeness in qualitative research reporting.

## Results

Twenty postpartum mothers who had recently given birth agreed to participate in this study. Participants varied in age, nationality, educational background, employment status, marital duration, mode of delivery, and parity. Table 1 presents descriptive data for participant characteristics.

**Table 1:** Socio-demographic characteristics of participants (*N* = 20)

Characteristic	*n*	%
Age		
18–24	4	20%
25–29	3	20%
30–34	3	15%
35–39	5	25%
40 years and above	4	20%
Nationality		
Saudi	17	85%
Non-Saudi	3	15%
Education		
Intermediate School	1	5%
High School	3	15%
Diploma	1	5%
Bachelor’s Degree	14	70%
Postgraduate (Master’s)	1	5%
Employment		
Employed	7	35%
Unemployed	11	55%
Student	2	10%
Marital Duration		
Less than 1 year	1	5%
1–3 years	7	35%
4–6 years	5	25%
7–10 years	4	20%
More than 10 years	3	15%
Mode of Delivery		
Vaginal delivery	7	35%
Caesarean section	11	55%
Assisted delivery (vacuum)	1	5%
Mixed delivery (VD & CS)	1	5%
Parity		
0 (Primiparous – first birth)	6	30%
1–2 births	8	40%
3–4 births	2	10%
5 or more births	4	20%
Total	20	100%

### Overview of emerging themes

Thematic findings revealed multiple dimensions shaping maternal recovery and satisfaction, including physical health, emotional well-being, breastfeeding support, newborn care education, access to health information, social support, hospital environment, birth experiences, pain management, discharge planning, and cultural practices. The structure of these themes and subthemes is presented in Table 2.

**Table 2:** Themes and subthemes emerging from interviews (*N* = 20)

	Themes	Subthemes
1	Physical Recovery and Childbirth Experience	Physical health; Pain management; Birth experience
2	Informational and Educational Support	Breastfeeding support; Newborn care education; Health information resources; Discharge experience
3	Emotional and social support	Emotional well-being; Family and social support; Lack of nursery support
4	Systemic and Cultural Context of Postpartum Care	Hospital environment and staff interaction; Cultural beliefs and practices

### Theme 1: physical recovery and childbirth experience

This theme corresponds to the maternal physical health domain of the WHO PNC Guidelines and describes women’s accounts of physical recovery, pain management, and childbirth experiences during the early postpartum period. Experiences varied by mode of birth and parity.

### Navigating physical recovery and pain management

Most women described physical pain and mobility following birth, and pain was generally well-controlled, with timely responses from nursing and medical staff. Regular monitoring and analgesia were commonly reported to facilitate early movement and basic self-care.

*“They used to check on me regularly and give me pain medication on time. I was able to move slowly and take care of my baby.” (PNC18*,* parity 0*,* VD)*.

In contrast, a few participants, most of whom had undergone caesarean birth, described experiencing more severe pain and restricted mobility. Some also reported delays in receiving pain relief.

*“I thought the pain would ease after a day or two… I wish someone had explained what to expect. This pain was much worse than vaginal delivery.” (PNC16*,* parity 6*,* CS)*.

Women differed in how pain affected mobility, rest, and newborn care, with greater difficulties more frequently described by those recovering from caesarean birth.

A participant described ongoing pain while being fully responsible for newborn care:

*“The pain was constant… I asked for stronger medication but no one came for a long time.” (PNC04*,* parity 5*,* CS)*.

### Birth experience: from reassurance to distress

Women described a wide range of birth experiences. Many reported positive experiences characterised by reassurance, respectful treatment, and responsive care during labour and delivery.

*“Despite the tear and bleeding*,* the doctors and nurses were kind and respectful. I felt genuinely cared for.” (PNC18*,* parity 0*,* VD)*.

Some participants, particularly first-time mothers, described feeling frightened during situations where urgent clinical decisions were made with limited explanation.

*“I was shocked when they suddenly told me I needed an emergency C-section. I felt scared and overwhelmed.” (PNC10*,* parity 0*,* CS)*.

Some women also described individual moments of emotional support during labour. Both primiparous and multiparous women reported such moments of emotional support.

*“The nurse held my hand and recited a prayer for me… It gave me the strength I needed.” (PNC14*,* parity 6*,* VD)*.

### Theme 2: informational and educational support

This theme corresponds to the postnatal education and health information domains of the WHO PNC Guidelines and describes women’s accounts of the information and guidance they received during the early postpartum period. Women frequently described postnatal information and education as fragmented and inconsistent. Instructions were often reported to vary between staff members and across shifts. Informational needs were more commonly described by primiparous women and those who had undergone caesarean birth, particularly in relation to newborn care and aspects of maternal recovery at the time of discharge.

### Breastfeeding guidance

Most women described breastfeeding guidance as the most structured and consistently delivered aspect of postnatal education. Women reported receiving organised antenatal sessions, followed by individualised, hands-on support from a lactation consultant after birth. Guidance commonly focused on latching, positioning, foods believed to support milk production, and the benefits of breastfeeding.

*“She explained everything clearly*,* how to position the baby*,* how to latch*,* what foods help with milk*,* and why breastfeeding is important for both of us.” (PNC19*,* parity 0*,* VD)*.

Several primiparous women described these encounters as reassuring after initial uncertainty.

*“At first*,* I was very scared to breastfeed because I thought it might affect my C-section and be dangerous for me. But when the lactation consultant stayed with me and explained everything step by step*,* I felt reassured and became confident that I could breastfeed safely.” (PNC17*,* parity 0*,* CS)*.

Women also described receiving electronic educational materials on their mobile phones, which they used to revisit the information while still in hospital.

*“They sent me the breastfeeding brochures on my phone*,* and I kept reading them again the next day.” (PNC08*,* parity 1*,* CS)*.

A few participants reported missing the initial visit of the lactation consultant due to sleep or being away from the room and assumed the consultant would return later.

*“She came while I was in the bathroom*,* and when I came back they told me she had already left. I thought she would pass by again the next day.” (PNC03*,* parity 3*,* CS)*.

### Fragmented or contradictory clinical guidance beyond breastfeeding

Women described their experiences with newborn care, maternal recovery, nutrition, and postnatal danger signs. In contrast to breastfeeding education, many women described inconsistent and fragmented information related to newborn feeding frequency, infant sleep, and aspects of maternal recovery. Conflicting advice from different staff members was commonly reported.

*“One nurse told me he is full and fine*,* and another said he is crying because he is still hungry. I didn’t know who to believe.” (PNC12*,* parity 0*,* VD)*.

These experiences were more frequently described by first-time mothers and women with medical complications.

*“Everyone told me something different*,* and because it was my first baby*,* I became very confused and anxious.” (PNC13*,* parity 0*,* CS)*.

Despite this, some women described individual nurses who provided clear and patient explanations.

*“One nurse sat with me calmly and explained everything slowly. That made a big difference for me.” (PNC09*,* parity 2*,* Vacuum-assisted VD)*.

Many women also reported minimal or absent education on maternal nutrition and postnatal danger signs, leading them to seek information from family members or online sources.

*“No one really explained what I should eat after delivery*,* so I just followed what my family told me and searched online.” (PNC10*,* parity 0*,* CS)*.

### Theme 3: emotional and social support

This theme aligns primarily with the interpersonal influences and health-promoting behaviours constructs of HPM and describes women’s accounts of emotional support, family involvement, and nursery support during the early postpartum period. Women described the early postnatal period as emotionally demanding. Experiences varied according to parity, mode of birth, and the availability of family support.

### Emotional vulnerability and the need for empathic care

Many women described feelings of anxiety, sadness, and fear during the first days after birth, even when their physical recovery was progressing well. Some women reported that staff recognised and responded to their emotional needs, while others described limited emotional engagement.

*“Physically I was healing*,* but inside I was overwhelmed… No one asked how I felt.” (PNC13*,* parity 0*,* CS)*.

Several women described individual moments of compassion from staff as emotionally reassuring, particularly during periods of concern related to their own or their baby’s condition.

*“My baby’s heart rate was very high… the nurse sat with me*,* listened to me*,* and offered a prayer for me.” (PNC06*,* parity 2*,* VD)*.

Some women also described personal spiritual practices, such as Qur’anic recitation, as sources of emotional comfort during labour.

*“When I recited Qur’an during labour*,* I felt a kind of peace that helped me tolerate the pain.” (PNC12*,* parity 0*,* VD)*.

In contrast, a small number of women described dismissive responses to emotional distress.

*“I told the nurse I was crying for no reason and she said*,* ‘It’s hormones.’’(PNC20*,* parity 2*,* VD)*.

One woman with a previous history of postnatal depression described fear of recurrence during her current postpartum experience.

*“After my previous delivery*,* I used to cry all the time without knowing why. I hated my baby for a long period and did not understand what was happening to me. Later*,* I realised it was postnatal depression. With this pregnancy*,* I am very afraid it might happen again.” (PNC15*,* parity 2*,* VD)*.

### Family presence as a source of emotional and practical support

Women with close family presence, particularly mothers or sisters, described receiving emotional reassurance and practical assistance with daily activities.

*“My mom stayed every night… she helped me shower and held the baby when I felt pain. Without her*,* I couldn’t have coped.” (PNC09*,* parity 2*,* Vacuum-assisted VD)*.

Such support was more frequently described as beneficial by primiparous women. In contrast, women without family nearby, including non-Saudi participants and those living away from relatives, reported loneliness and emotional strain.

*“I’m not from Riyadh and my sister couldn’t come yesterday… At night it was just me with the baby*,* I was in pain*,* and I felt scared and unsure what to do.” (PNC02*,* parity 3*,* CS)*.

Some multiparous women described expecting additional institutional support due to previous hospital experiences and reported difficulty adjusting when this was not available.

### Lack of nursery support and emotional strain

Participants reported the absence of nursery services as a difficulty during hospitalisation. This was more frequently described by women recovering from caesarean birth, mothers of twins, and women experiencing fatigue or complications.

*“After my C-section*,* I could barely move… I asked if there was any nursery service*,* and they said no.” (PNC16*,* parity 6*,* CS)*.

Mothers of twins described the continuous responsibility for both infants as physically and emotionally demanding.

*“I had twins and was completely exhausted… I felt like I would collapse.” (PNC11*,* parity 1*,* mixed VD/CS)*.

Some women also described technical barriers, such as non-functional call buttons, which delayed assistance. These experiences were more frequently described by women recovering from caesarean birth and mothers of twins.

*“I kept pressing the call button… later I found out it wasn’t working. I really needed someone to help me even for a short while.” (PNC02*,* parity 3*,* CS)*.

### Theme 4: systemic and cultural context of postpartum care

The WHO Postnatal Care Guidelines and the situational and interpersonal influences constructs of HPM. It describes women’s accounts of hospital-related factors and cultural practices that shaped their postpartum experiences. Women described the hospital environment, staff interactions, institutional routines, and cultural traditions as influencing their daily experiences during hospitalisation and the immediate post-discharge period.

### Hospital environment and staff interaction

Most women described nurses and physicians as respectful, compassionate, and responsive, particularly in relation to breastfeeding support, newborn hygiene, and routine monitoring. Regular checks and clear explanations were commonly reported. Some women also described being transferred to private rooms or receiving additional consideration for rest.

*“After my C-section… I was very exhausted. The nurse helped move me quickly to a private room on the second day*,* and being able to rest in peace really made a difference.” (PNC01*,* parity 1*,* CS)*.

In contrast, a smaller number of women reported concerns related to room cleanliness, delayed assistance, or fragmented communication.

*“My bed sheets had stains from the previous patient… I asked to change them*,* but no one came for hours.” (PNC04*,* parity 5*,* CS)*.

Some women also described rotational staffing as disrupting continuity of care.

*“Each nurse asked me the same things again… It felt like they didn’t read my file.” (PNC05*,* parity 5*,* CS)*.

#### Hospital physical environment and NICU communication

Women described the hospital environment in relation to privacy, noise, and opportunities for rest. Focusing on the physical environment and interdepartmental communication rather than bedside care. Those in private rooms reported greater privacy and fewer disturbances.

*“When they moved me to a private room*,* I finally felt calmer and could rest. It really changed how I felt.” (PNC15*,* parity 2*,* VD)*.

In contrast, women in shared rooms described the environment as crowded and noisy.

*“The room was very crowded and noisy. I couldn’t sleep at all*,* and that made me feel even more exhausted emotionally.” (PNC05*,* parity 5*,* CS)*.

A small number of women described difficulties in communication between the postnatal ward and the neonatal intensive care unit (NICU). One mother of a premature infant reported limited updates and the absence of a direct contact point with the NICU.

*“They took my baby to the NICU*,* and when I asked about him*,* no one here could tell me anything… there was no number to call. They told me*,* ‘You have to go yourself.’ My husband pushed me in the wheelchair so I could see my baby.” (PNC07*,* parity 1*,* CS)*.

Another woman described being transferred to an observation unit while her newborn remained in the postnatal room.

*“When my blood pressure became high*,* they transferred me… later my sister found my baby alone in the room.” (PNC17*,* parity 0*,* CS)*.

### Cultural traditions shaping postpartum practices

Women described cultural traditions as influencing diet, rest, and caregiving during the early postpartum period. Many Saudi and Arab mothers referred to the Al-Nifas 40-day rest period and the involvement of family members in providing food and infant care.

*“In our tradition*,* we rest for forty days… My mom cooks for me*,* helps with the baby*,* and makes fennel tea. It really helps.” (PNC08*,* parity 1*,* CS*,* Saudi)*.

Some women described differences between family advice and clinical recommendations, particularly regarding diet after caesarean birth.

*“My family told me not to eat any kind of meat after my C-section because they believe it can cause complications. The nurse said it was completely fine*,* but I felt torn between what my mother insisted on and what the hospital advised.” (PNC01*,* parity 1*,* CS*,* Non-Saudi)*.

Some women reported questioning traditional practices and prioritising medical advice.

*“My relatives had a lot of advice*,* but I chose to follow what the doctors said.” (PNC17*,* parity 0*,* CS)*.

Faith-based practices, including trust in God (Tawakkul), were also described by women across different parity and birth modes as sources of reassurance.

*“I kept telling myself that everything is in Allah’s hands*,* and that thought gave me strength.” (PNC03*,* parity 3*,* CS)*.

### Navigating conflicting information across systems and cultures

Some women reported that this overlap led to confusion and uncertainty, particularly among first-time mothers and those with medical complications.

*“Everyone was telling me something different… because it was my first baby and my delivery was complicated*,* I felt lost.” (PNC10*,* parity 0*,* CS)*.

Some women described inconsistent messages from healthcare providers themselves.

*“One nurse told me to wake him every two hours*,* and another said I should let him sleep.” (PNC13*,* parity 0*,* CS)*.

Several women reported relying on family traditions or online information when clinical guidance was perceived as unclear.

*“When I didn’t get a clear answer at the hospital*,* I went back to my mother’s advice and searched online.” (PNC11*,* parity 1*,* mixed VD/CS)*.

## Discussion

This study provides an interpretive understanding of how postpartum women in Saudi Arabia experienced physical recovery, emotional adjustment, informational needs, and culturally shaped expectations. Although clinical care was generally viewed as competent, gaps in emotional support, education, and systemic resources shaped women’s confidence, engagement in newborn care, and perceived safety.

In this study, women’s birth experiences varied markedly between vaginal and caesarean delivery and had a significant influence on physical recovery, emotional interpretations of childbirth, and readiness for motherhood. Participants who experienced vaginal birth described quicker recovery and greater functional independence despite fear and intense labour pain. In contrast, caesarean birth was linked to more severe postoperative pain, restricted mobility, delayed recovery, and heightened emotional vulnerability. These findings are consistent with international evidence showing slower physical recovery after caesarean birth [19, 20].

Our study showed that the absence of nursery support and limited overnight assistance intensified postpartum challenges. Within HPM, nursery unavailability represents a situational constraint that reduces perceived control, undermines self-efficacy, and limits women’s ability to engage confidently in health-promoting behaviours [13]. The lack of nursery services aligns with the Mother-and-Baby-Friendly Maternity Care Initiative, which promotes continuous rooming-in [10]. However, while rooming-in supports mother–infant bonding and breastfeeding, the findings indicate that inflexible implementation, without consideration of women’s physical capacity or available support, may unintentionally intensify exhaustion and psychological distress, particularly following caesarean birth [21].

Social and family support emerged as a central protective factor shaping women’s emotional stability, confidence, and coping capacity during the early postpartum period. Interpersonal influences, particularly emotional reassurance, practical assistance, and shared experience, strengthened maternal self-efficacy and supported engagement in newborn care [13]. Women with overnight support from mothers or sisters reported feeling safer, more rested, and better able to manage newborn care. These findings reflect quantitative evidence showing that partner involvement predicts postpartum quality of life and well-being [22], and that higher perceived social support is associated with better maternal functioning and mother–infant attachment [23].

Staff interaction emerged as a key influence shaping women’s emotional security, confidence, and trust in care. Most participants described nurses and physicians as respectful, compassionate, and responsive. These behaviours enhanced women’s sense of safety and satisfaction, consistent with international evidence showing that respectful, empathetic maternity care improves emotional well-being and overall care experiences [24, 25].

Conversely, some participants experienced dismissive or rushed interactions from staff that intensified feelings of fear and isolation. Evidence shows that disrespectful care undermines women’s dignity and emotional security [7], while inadequate emotional support is associated with poorer psychological outcomes and reduced self-efficacy [7, 26]. In this context, such interactions may reflect broader systemic pressures, including high workloads, staff rotation, and competing clinical demands. These findings are consistent with established principles of respectful, person-centred maternity care, highlighting the central role of dignity, compassion, and responsive communication, and underscore how emotional support shapes women’s perceptions of safety and control during the early postpartum period [1].

Inconsistent and fragmented postpartum education emerged as a major gap in care, contrasting with WHO PNC Guidelines, which emphasise structured and consistent instruction on breastfeeding, newborn care, maternal self-care, and danger signs [1]. Limited guidance, particularly among first-time mothers, contributes to uncertainty and reduced confidence as women prepare for discharge [27–29].

The hospital’s physical environment also shaped women’s comfort, and emotional adjustment. Women in private rooms consistently reported greater privacy, better sleep, and improved psychological comfort, whereas shared rooms were described as noisy, crowded, and lacking privacy. These experiences align with evidence showing that ward noise, overcrowding, and reduced privacy disrupt maternal sleep and contribute to fatigue and emotional strain [30–33].

Women’s readiness for discharge differed by mode of birth, maternal experience, and available support. Multiparous women with vaginal births generally felt prepared once clinical stability was confirmed, whereas many women recovering from caesarean birth and most first-time mothers felt physically and emotionally unprepared and anxious. These experiences are consistent with evidence linking caesarean birth and limited postpartum education to lower discharge readiness and increased post-discharge anxiety [20, 26, 34]. Reassurance was associated with attentive nursing care and the presence of supportive family members during the transition home [35]. However, discharge education was inconsistently provided, with limited guidance on maternal self-care, neonatal danger signs, and essential newborn care, reflecting a broader tendency to prioritise clinical stability over holistic preparedness [26, 27]. Given that effective discharge requires emotional readiness, adequate information, and continuity of support [1], these gaps indicate a need for more structured discharge education in the early postnatal period.

Previous research has shown that cultural beliefs and postpartum traditions play an important role in shaping women’s emotional adjustment and perceptions of recovery [36, 37]. Consistent with this evidence, findings from the present study indicate that practices such as Nefas often enhanced women’s sense of emotional security and family cohesion during the early postpartum period. However, participants also described that rigid enforcement of these traditions or tension between family expectations and clinical advice could create stress and undermine their sense of autonomy. This dual role of cultural practices, providing emotional support while at times generating decisional conflict, has also been reported internationally [38], highlighting the importance of person-centred postpartum care that balances family involvement with women’s autonomy. In addition, spiritual and religious practices, including prayer, Qur’anic recitation, and reliance on God, emerged from participants’ accounts as key emotional coping strategies, consistent with global evidence linking spirituality to enhanced psychological resilience during the perinatal period [39].

### Implications for policy, practice, and future research

The findings highlight several areas that healthcare systems may consider when strengthening postpartum care, particularly in relation to informational continuity, emotional support, and discharge preparedness. Women valued clear communication, timely breastfeeding support, and structured guidance, suggesting a need to enhance postpartum education across antenatal, inpatient, and post-discharge phases. Expanding access to lactation support and improving discharge preparation may help ensure women feel emotionally and practically ready for home care. Flexible support strategies for women recovering from caesarean birth or those without companions may also help balance rooming-in practices with maternal recovery needs. Digital follow-up tools and nurse-led postpartum clinics may further support continuity of care, aligning with broader national priorities for women’s health and digital health integration [40].

The findings also reinforce the importance of respectful, person-centred communication throughout labour, recovery, and discharge. Brief emotional check-ins, sensitivity to individual differences in pain and mobility, and culturally appropriate explanations may strengthen maternal confidence and perceived capacity during the early postpartum period.

Future research could explore postpartum experiences across multiple institutions and regions to enhance transferability. Comparative studies between private and public hospitals are essential for understanding variations in care delivery and patient experiences. In addition, studies involving partners, family members, and healthcare providers may offer deeper insight into support dynamics. At the same time, examination of system-level factors such as staffing patterns and workload could help explain persistent informational and emotional gaps in care.

### Limitations

This study was conducted in a single tertiary hospital in Riyadh, which may limit the transferability of the findings to other contexts within Saudi Arabia or beyond. Additionally, all interviews were conducted during hospitalisation, a period characterised by heightened emotions, physical discomfort, and fatigue, which may have introduced recall bias or shaped how women articulated and interpreted their experiences. Despite these limitations, the findings offer rich, contextually grounded insights into postpartum care experiences among a diverse group of women.

As with all qualitative research, the interpretation of the findings may have been influenced by the researcher’s positionality. The first author’s background as a Saudi health educator with extensive experience in patient education may have shaped the emphasis on gaps in informational continuity, emotional support, and discharge preparedness. Familiarity with local cultural norms supported contextual understanding of participants’ accounts, while being external to the study hospital may have influenced the interpretation of institutional practices and system-level constraints. To mitigate potential bias, analytic decisions were discussed collaboratively with a senior academic co-author, with reflexive dialogue used throughout the analysis.

## Conclusion

This study highlights how physical recovery, emotional well-being, social support, and institutional practices shape women’s early postpartum experiences in a Saudi tertiary hospital. While clinical care was generally viewed as competent, gaps in emotional support, structured education, nursery availability, and discharge preparedness affected women’s confidence and sense of safety during the postpartum transition. The findings emphasise that high-quality postpartum care extends beyond clinical stability to include responsive communication, consistent education, and flexible support. Although grounded in a single setting, the study identifies key areas for strengthening postpartum services and supports the value of integrated, woman-centred approaches to enhance maternal well-being and quality of care.

## Data Availability

The datasets generated and/or analyzed during the current study are available from the corresponding author upon reasonable request.

## Abbreviations

COREQ: Consolidated Criteria for Reporting Qualitative Research
CS: Caesarean section
HPM: Pender’s Health Promotion Model
KKUH: King Khaled University Hospital
NICU: Neonatal intensive care unit
PNC: Postnatal care
RTA: Reflexive Thematic Analysis
VD: Vaginal delivery
VD/CS: Mixed mode of delivery (vaginal delivery and caesarean section)
WHO: World Health Organization
WHO PNC Guidelines: World Health Organization Postnatal Care Guidelines
